# Effects of Mustard Gas on the Eyes

**Published:** 1918-10

**Authors:** 


### OPHTHALMOLOGY AND OTO-LARYNGOLOGY

---

***Effects of Mustard Gas on the Eyes.*** By George S. DERBY, M. O. R. C., U. S. A. No. 13, General Army Hospital B. E. F., U. S. Army Base Hospital N° 5.

It is important that our ophthalmologists should be well-informed as to the effects of mustard gas and be thereby in a position to assist their medical and surgical confreres when asked for advice.

About 80 o/o of the cases received show eye lesions of greater or less severity. The lesions are of the nature of a chemical burn of the skin of the lids, conjunctiva, and of the cornea in a considerable percentage.

*The Lids.* These are reddened, swollen, and often show humorous large bullae; they are kept tightly closed. The irritation and photophobia are so intense that not infrequently resort must be had to one or two drops of cocaine and the use of a lid elevator in order to make a proper examination of the eye. Separation of the lids is often followed by a gush of tears.

In mild cases there is slight conjunctival infection with slight photophobia, but in severe cases the infection is severe with at times a very marked white edema and chemosis of the membranes, most noticeable in the area of the palpebral fissure. A secondary infection of the conjunctiva following gassing is not at all uncommon, and ciliary infection is frequent. In the more severe cases, careful examination will nearly always reveal a certain amount of corneal involvement, varying from a slight roughening of the epithelium to a well-defined opacity, usually in the form of a band extending across the cornea in the area corresponding to the palpebral fissure. In certain rare instances, probably where some of the fluid has come into direct contact with the cornea, this band assumes an almost porcelain whiteness.

Ulceration of the cornea is rare. The writer has seen but three cases with a definite loss of substance. According to the French these corneal lesions are stained by fluorescein. This also is rare. Sometimes as a result of corneal irritation, it may be necessary to use atropin three or four times a day to secure a satisfactory dilatation of the pupil, but in most instances, a drop of a one per cent. solution twice a day will prove satisfactory.

The intense photophobia, which occurs so frequently, may be explained by the corneal involvement,

*Prognosis.* Cerise divides these cases into three groups :

1. Benign, representing 10 to 15 o/o of cases. Duration 10 to 15 days.

2. Medium, about 80 o/o of the cases. Duration 5 to 6 weeks.

3. Severe, 3 to 5 o/o, seen in those with marked general symptoms, who often develop a broncho-pneumonia.

The larger proportion of the cases might be placed under Class 1.

Speaking entirely from the ocular standpoint, it may be estimated that nearly a third of the cases may be discharged to duty in a week, a third more at the end of the second week. Perhaps a fifth of the cases require three to four weeks, and the remainder as much as two months. With very rare exceptions complete recovery results.

*Treatment.* The eye should be protected from light, but not bandaged. Satisfactory eye shades can be manufactured from three or four thicknesses of blue paper. The eyes should be gently irrigated in severe cases every three or four hours with some bland solution, such as normal, saline, soda bicarbonate 1 o/o, or boric acid, best heated to about 100 F before using. Liquid paraffin may be instilled later, or in the later stages argyrol in a 25 o/o solution is found to be soothing.

Castor oil has proved to be more irritating than liquid paraffin.

Atropin should be used in cases of corneal involvement, and a one per cent. solution instilled twice a day will usually be found to keep the pupil well dilated. Except in cases, the use of atropin may be discontinued after two or three days.

Cocaine should not be used as a routine, but is valuable for the first examination of the eye to relax the spasm of the lids.

Where hyperemia of the conjunctiva exists for a long time, a mild astringent collyrium may be used.

When the cornea clears and the infection begins to disappear, it is very important to get these patients up and accustom them to a greater degree of illumination.

